# Effect of soil spatial configuration on *Trifolium repens* varies with resource amount

**DOI:** 10.1371/journal.pone.0263290

**Published:** 2022-01-31

**Authors:** Yi-Wen Pan, Zhi-Xia Ying, Michael P. Nobis, Anna M. Hersperger, Chen Shi, Gang Ge

**Affiliations:** 1 Key Laboratory of Poyang Lake Environment and Resource Utilization, Ministry of Education, School of Environmental & Chemical Engineering, Nanchang University, Nanchang, China; 2 Swiss Federal Institute for Forest, Snow and Landscape Research WSL, Birmensdorf, Switzerland; 3 School of Life Sciences, Nanchang University, Nanchang, China; Seoul National University, REPUBLIC OF KOREA

## Abstract

Soil spatial heterogeneity involves nutrients being patchily distributed at a range of scales and is prevalent in natural habitats. However, little is known about the effect of soil spatial configurations at the small scale on plant foraging behavior and plant growth under different resource amounts. Here, we experimentally investigated how a stoloniferous species, *Trifolium repens*, responded to varied resource amounts and spatial configuration combinations. Plant foraging behavior (i.e., the orientation of the primary stolon, mean length of the primary stolon, foraging precision, and foraging scale) and plant growth (i.e., total biomass, root biomass, shoot biomass, and root/shoot) were compared among differently designed configurations of soil resources in different amounts. The relationships of foraging behavior and plant biomass were analyzed. The results showed that the effect of the spatial configuration of soil resources on *Trifolium repens* depended on the resource amount. Specifically, when the total resource amount was low, fragmented soil patches promoted root foraging and increased *Trifolium repens* plant biomass; however, when the total resource amount was high, the soil spatial configuration did not affect foraging behavior or plant growth. Our results also showed that plant growth was facilitated by root foraging scale to adapt to low resource amounts. We conclude that the spatial configuration of soil resources at small scales affects whole plant growth, which is mediated by a distinct foraging strategy. These findings contribute to a better understanding of how the growth strategy of clonal plants responds to heterogeneous environments caused by different resource amounts and its spatial configurations.

## Introduction

Plant growth depends on the available resources, such as soil nutrients, water, and light [1–3]. At the microsite scale, soil resources are often distributed heterogeneously [4, 5], showing different amounts of soil resources and spatial configurations. Plants, especially clonal plants, have developed foraging behavior by plastic adjustments in foraging organs such as root systems and spacers (e.g., stolons or rhizomes) to cope with heterogeneous soil patches [6–8]. They can, for example, increase root proliferation and place more ramets in nutrient-rich patches [3, 9, 10], and increase specific root length in nutrient-poor patches [11]. These foraging behaviors can improve the ability of plants to capture resources efficiently [12].

Plant foraging behavior is affected by the spatial configuration of the supply of resources [9, 13]. Soil spatial configuration is commonly referred to as the soil patch size of their location [14]. For instance, with the same supply amount of soil resources, some researchers found that root proliferation in nutrient-rich patches was more significant in large patch treatments than in small patch treatments [13, 15, 16]. Under competition, root proliferation in nutrient-rich patches was observed in both the large patch treatment and small patch treatment; when individual plants grew alone, foraging behavior was observed only in the large patch treatment [17]. This finding indicates that the availability of resource amount for plants might affect plant foraging behavior in spatial configurational soil habitats since competition mainly limits the availability of resource amount for individual plants [18]. However, current studies on the effects of the spatial configurations of soil resources on plants have been conducted irrespective of the amount of soil resources. How individual plants respond to soil spatial configurations under varying total resource amounts remains largely unexplored.

Considering different resource amounts when studying the response of plants to changes in spatial configurations in heterogeneous soil is necessary for several reasons. First, resource amount needed for plant growth is very important because it can determine root proliferation, below- and aboveground allocation, plant productivity and plant distribution [19–22]. Second, previous studies have shown that there are joint effects of soil heterogeneity and nutrient availability on plant assemblages [23, 24]. For some species, in comparison to the main effect of resource amount, the effect of resource amount interacting with soil heterogeneity on plant growth is greater [24]. For example, it has been shown that the proportion of *Lolium perenne* aboveground biomass was affected by the joint effects of nutrient availability and heterogeneity but not by nutrient availability alone [24]. However, these studies are focused on plant assemblages, and they failed to elucidate the root growth of individual species in response to soil heterogeneity under different resource amounts.

Although foraging behavior has often been found to occur in heterogeneous soil, its roles in plant growth remain unclear [25–27]. Some studies have shown that increasing root proliferation and placing more ramets in nutrient-rich patches could promote the amount and efficiency of resource capture by these ramets, thus increasing their growth [15, 16, 26]. Moreover, due to clonal integration (i.e., resource sharing between ramet systems), their efficiency in capturing resources in nutrient-rich patches could also promote the growth of the whole plant [28–30]. However, other studies found no benefits from foraging behavior for plant growth [17, 27, 31]. A possible reason for the inconsistency in these results is that the resource amount may also affect the importance of foraging behavior in plant growth. For example, Sammul et al. (2011) found that the advantage of foraging behavior (i.e., long spacers) is weaker in nutrient-rich conditions than in nutrient-poor conditions [6]. Moreover, the measurements of foraging behavior used in such studies are often different and difficult to compare.

To assess the effect of soil heterogeneity in terms of both amount and configuration of soil resources on foraging behavior and plant growth and to explore the importance of different foraging behaviors for the growth of the whole plant, we designed an experiment with *Trifolium repens* grown in six different patterns of heterogeneous soil (i.e., three different soil spatial configurations with two different resource amounts). Specifically, we tried to answer the following questions: (1) Does the effect of the spatial configuration of soil resources on foraging behavior and on plant growth depend on the total resource amount? (2) Does plant growth benefit from foraging behavior? If so, which foraging behavior?

## Materials and methods

### Species

*Trifolium repens* L. (Fabaceae) is a common stoloniferous herbaceous perennial species [32]. It is a clonal plant consisting of ramets (or its repeating modules) connected by stolons [32]. *Trifolium repens* is not only a valuable forage legume in pastures and intensively managed temperate grasslands, but it also contributes as a robust clonal species to city greening [33]. We chose *Trifolium repens* as our study species because it is a clonal plant that is characterized by a plastic structure, and its performance is linked to heterogeneous environments and resource availability [34, 35]. Moreover, its aboveground stoloniferous organs allow us to record its aboveground foraging behavior.

### Experimental design

**Fig 1:**
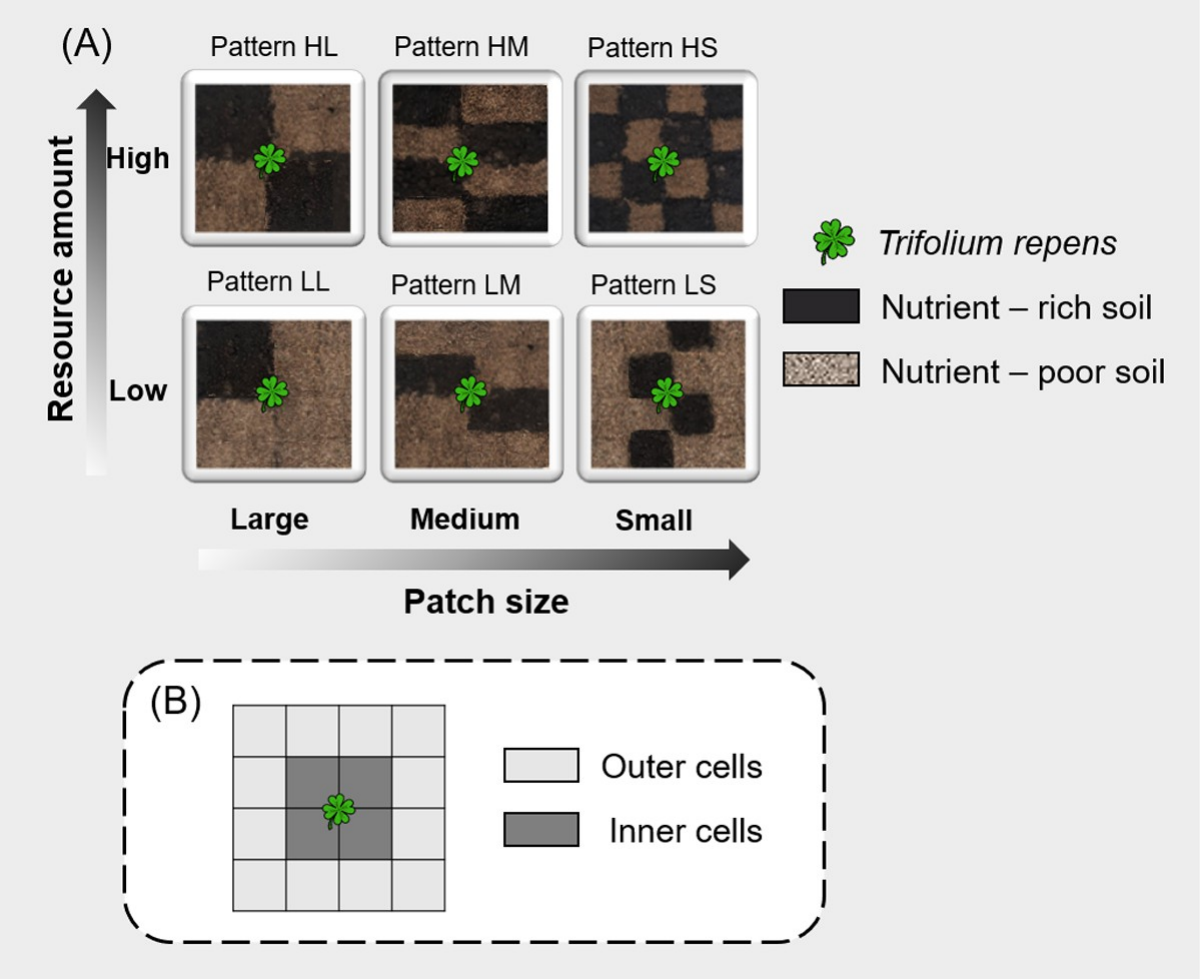
Schematic representation of the experimental design. In (A): Each pot (4 × 4 cells) represents a unique pattern determined by different resource amounts and spatial configurations. In (B): the light grey cells can be described as outer cells, and the dark grey cells can be described as inner cells.

The experiment was carried out on the rooftop of the Life Science building of Nanchang University, Nanchang, China. Six combinations of two resource amounts (i.e., low and high resource amounts) and three spatial configurations were formed based on the arrangement of the potting soil and vermiculite in 41 × 41 × 8 cm^3^ (length × width × height) plastic pots (Fig 1A). The potting soil and vermiculite can be treated as nutrient-rich and nutrient-poor soil, respectively. Both of them were bought from commercial suppliers. Information on the soil characteristics information was provided by the manufacturer. For each 50 L potting soil, organic substance ≥ 65%, N+P2O5+K2O ≥ 5%, pH = 5.5–6.5. We did not measure the characteristics of vermiculite because it is a well-known nutrient-poor substrate [36]. The resource amount was characterized by altering the percentage of nutrient-rich soil; i.e., a low resource amount contained 25% nutrient-rich soil and a high resource amount contained 50% nutrient-rich soil. Different spatial configurations of resource amounts were characterized by altering the patch size (or patch number) (Fig 1A). The smaller the patch size is, the more fragmented the resource amount is. To create these six heterogeneous patterns, each pot was divided into 4×4 cells using plastic plates before adding these substrates, and after adding the substrates we slowly pulled out the plastic plates. Thus the 16 soil cells in each plot were interconnected, and roots could grow freely throughout the whole pot. Twelve replicates were provided for each pattern and a total of 72 pots were established. At the end of April 2019, 72 seedlings that were approximately ten days old and of similar size were planted into the center of each pot. The pots were randomly placed to eliminate the influence of other factors such as light on the results. The quantity (1.5 L per pot) and frequency (once every 3–4 days) of watering for the 72 pots were controlled, and water was irrigated gently and slowly in each pot to avoid disturbing the soil. Weeds were regularly removed from all pots. Photo-ecology agricultural films fixed with a plastic frame were used to cover the experiment on rainy days.

### Harvest and measurements

The experiment ended after eight weeks when *Trifolium repens* almost completely covered the patterns. We harvested the aboveground parts (including leaves, petioles, stolons, and inflorescences) and root parts located in nutrient-rich cells and nutrient-poor cells separately in each pot. Length and number of stolons were measured. Both shoots and roots were carefully washed to remove all soil. Finally, all shoots and roots were oven-dried at 80°C for 48 h and weighed.

Four foraging traits were used: orientation of the primary stolon (proportion of the primary stolons growing toward the nutrient-rich patches), mean length of the primary stolon, foraging precision, and foraging scale [37–39]. Foraging precision measures the proportion of plant roots in nutrient-rich patches [7, 27]. Foraging scale is generally measured using a proxy such as root length, total surface area, breadth, or biomass [7]. The more extensive the rooting is, the more biomass found in outer cells and the larger the foraging scale. Thus, the foraging scale was calculated as the ratio of root biomass per outer nutrient-rich cell to root biomass per inner nutrient-rich cell (Fig 1B).

Four growth traits were calculated: total biomass, root biomass, shoot biomass (aboveground biomass), and the ratio of root biomass to shoot biomass (root/shoot).

### Data analysis

T-test or Wilcoxon rank-sum test (for data with non-normal distribution) were used to test the overall difference in plant foraging behavior and growth between high and low resource amounts. In this step, we disregarded the soil spatial configuration.

One-way analysis of variance (ANOVA) or Kruskal-Wallis H test (when ANOVA assumptions were not met) was used to test the effect of soil spatial configuration on plant foraging behavior and growth under high and low resource amounts. ANOVA results do not provide detailed information regarding the differences among configurational groups. Therefore, post-hoc multiple comparisons were used to clarify the differences between particular pairs of configuration groups under the same resource amount. In addition, multiple comparisons are usually accompanied by an increased type I error issue [40], and it is necessary to adjust the P value accordingly. Therefore, for post-hoc multiple comparisons, pairwise t-test (after ANOVA) and pairwise Wilcoxon rank-sum tests (after Kruskal-Wallis H test) with Benjamini-Hochberg adjustment were used. We chose the Benjamini-Hochberg adjustment because it is one of the most commonly used methods, and it not only reduces false positives, but also minimizes false negatives [41], and increases the reliability of the results.

To explore the effect of foraging behavior on whole plant growth at low and high resource amounts, multiple linear regression was used to test the relationship between foraging behavior variables and total plant biomass. All the foraging behavior variables were standardized to a mean of 0 and a standard deviation of 1, therefore the relative importance among foraging behavior variables could be directly compared. The variance inflation factor (VIF) of each foraging behavior variable was lower than 2, so multicollinearity was not a problem. All above statistical analyses were performed using R 4.0.3 for Windows [42].

## Results

Overall, the orientation of the primary stolon (Wilcoxon test, p = 0.773), mean length of the primary stolon (t-test, p = 0.457), foraging precision (Wilcoxon test, p = 0.222), foraging scale (Wilcoxon test, p = 0.205), total biomass (Wilcoxon test, p = 0.723), root biomass (Wilcoxon test, p = 0.218), and shoot biomass (Wilcoxon test, p = 0.496) did not differ significantly between the groups with high and low resource amounts. However, the root/shoot was significantly higher at low resource amounts than at high resource amounts (Wilcoxon test, p = 0.011).

**Fig 2:**
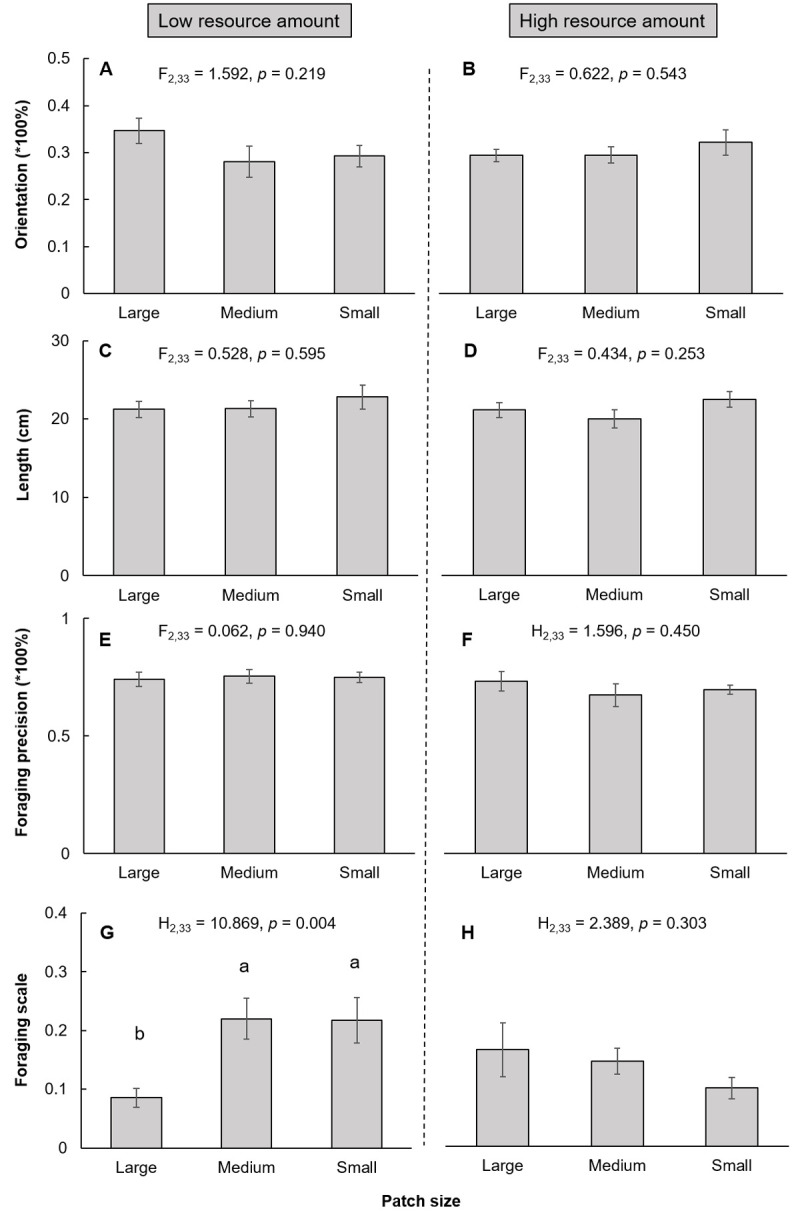
Effect of soil spatial configuration on plant foraging behavior (mean ± SE) under low and high resource amounts. (A) and (B): orientation of the primary stolon. (C) and (D): length of the primary stolon. (E) and (F): foraging precision. (G) and (H): foraging scale. Bars sharing the same letters or no letters above indicate that there are no significant differences (p > 0.05), while different letters indicate that there are significant differences among these treatments (p < 0.05).

**Fig 3:**
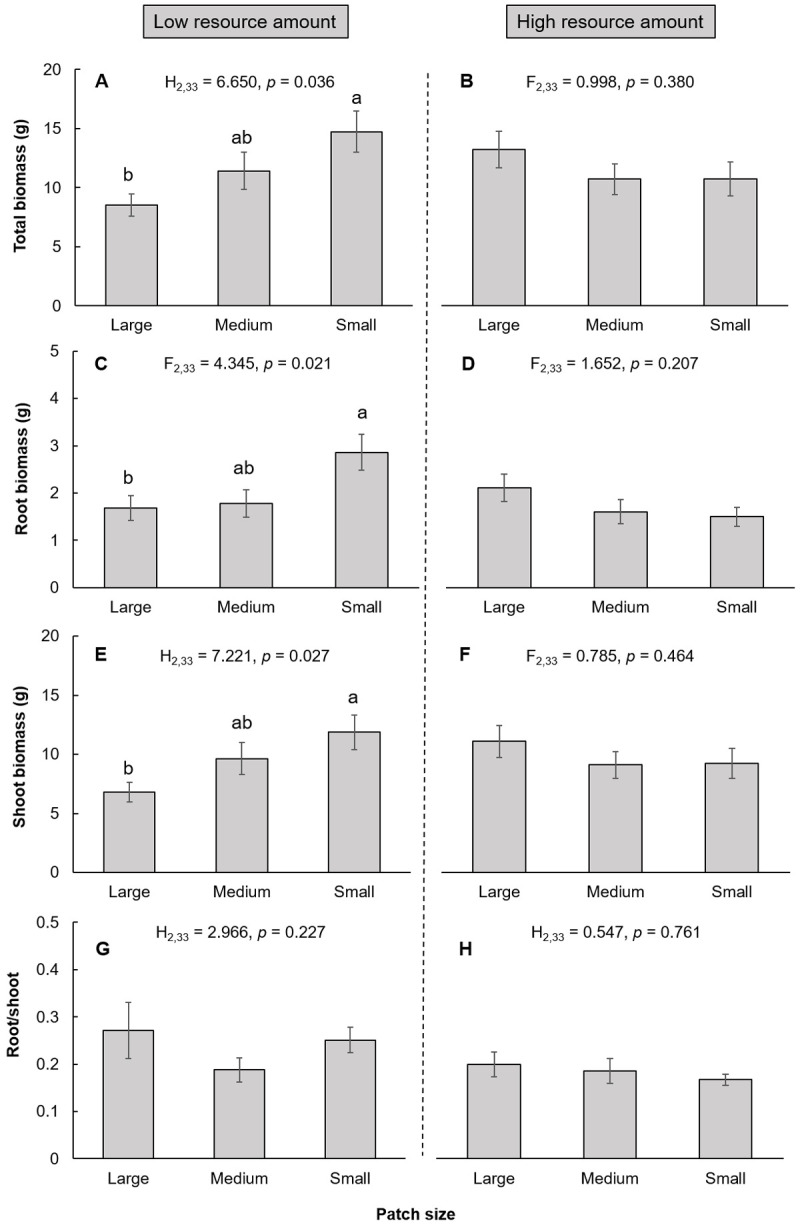
Effect of soil spatial configuration on plant growth (mean ± SE) under low and high resource amounts. (A) and (B): total biomass. (C) and (D): root biomass. (E) and (F): shoot biomass. (G) and (H): root/shoot. Bars sharing the same letters or no letters above indicate that there are no significant differences (p > 0.05), while different letters indicate that there are significant differences among different patterns (p < 0.05).

The effect of soil spatial configuration on plant behavior and growth depended on the resource amount (Figs 2 and 3). Under low resource amounts, the soil spatial configuration had a significant effect on the foraging scale (Fig 2G), total biomass (Fig 3A), root biomass (Fig 3C), and shoot biomass (Fig 3E), but had no significant effect on the orientation of the primary stolon, mean length of the primary stolon, foraging precision or root/shoot (Figs 2A, 2C and 3E, 3G). The foraging scale, plant total biomass, root biomass, and shoot biomass were significantly greater in the small patch patterns than in the large patch patterns (Figs 2G and 3A, 3C and 3E). However, under high resource amounts, the soil spatial configuration did not significantly affect neither plant foraging behavior nor plant growth (Figs 2 and 3).

Overall, foraging behavior explained plant growth better under low resource amounts than under high resource amounts (Table 1). Foraging scale had the strongest positive impact on plant total biomass at low resource amounts but not at high resource amounts. The mean length of the primary stolon had a marginally positive effect on plant total biomass, while the orientation of the primary stolon and foraging precision had no significant effect on plant total biomass at both low and high resource amounts (Table 1).

**Table 1:** Effect of plant foraging behavior on plant total biomass.

	Low resource amount				High resource amount			
Variable	Estimate	SE	*t*	*p*	Estimate	SE	*t*	*p*
Intercept	11.000	0.879	12.520	**<0.001**	11.755	0.826	14.232	**<0.001**
Orientation of the primary stolon	-0.661	0.708	-0.933	0.358	-1.079	1.011	-1.068	0.294
Mean length of the primary stolon	1.656	0.813	2.038	0.050	1.551	0.906	1.713	0.097
Foraging precision	0.358	0.879	0.308	0.761	0.533	0.779	0.684	0.499
Foraging scale	2.069	0.869	2.381	**0.024**	0.177	0.996	0.177	0.680
R^2^	0.322				0.147			

Values of p < 0.05 are in bold.

## Discussion

We found that a lower resource amount led to a higher root/shoot irrespective of soil spatial configuration, suggesting that biomass partitioning is adjusted to equalize the limitation of resource amount on plant growth [22]. Developing aboveground parts is more costly than developing belowground parts, and thus, plants allocate more biomass into roots when the resources are low [43].

Our results indicate an interesting phenomenon that the effect of spatial configuration depends on resource amount, leading to diverse effects of soil spatial configurations on plant foraging behavior and growth. This result is in line with that of Maestre and Reynolds (2007), who found that plant assemblages had more biomass and root proliferation in heterogeneous soil than in homogeneous soil, and these differences increased with increased nutrient availability [24]. However, they did not investigate plant responses to different spatial configurations of soil heterogeneity and failed to distinguish the root foraging behavior of individual plants under different resource amounts. In our case, under low resource amounts, fragmented spatial configuration positively affected the foraging scale and plant biomass, indicating that the fragmented spatial configuration facilitates root foraging extensively. Plants tend to promote nutrient capture by developing extensive root systems [38, 44]. Some nutrient elements are movable, such as nitrogen (N), and some elements are immobile in the soil such as phosphorus (P) and potassium (K). In comparison to a clustered soil configuration, a fragmented spatial configuration results in more dispersed nutrient distribution due to spatial autocorrelation, thus enhancing the opportunity for an individual plant with a large foraging scale to uptake multiple nutrients and lead to greater whole plant growth [45]. However, the soil spatial configuration had a neutral effect on plant behavior and growth under high resource amount conditions. One possible reason is that when the resource amount is sufficient, plants do not simulate forage extensively, and plant growth does not differ in different soil spatial configurations. Similar results were found in the clonal plants *Buchloe dactyloides* and *Hydrocotyle vulgaris* when grown under 50% resource conditions [17, 31]. In contrast, soil spatial configuration significantly affected *Glechoma Hederacea* when the resource amount was 50%, and greater biomass was produced in clustered soil configurations than in fragmented spatial configurations. These results indicate that the effect of soil spatial configuration in heterogeneous environments also differs among species [14].

In our study, the orientation of the primary stolon and mean length of the primary stolon did not significantly differ among the different resource amounts or soil spatial configurations. This result may be because roots are the component of the clonal growth form that is most responsive in terms of foraging for nutrients in soil patches [15], whereas the aboveground parts of clonal plants, such as stolons and branches, mainly respond to heterogeneous light conditions [43]. In our study, light was a homogeneous resource.

The foraging behavior explained plant growth better at low resource amount than at high resource amounts, and plant growth was positively correlated with root foraging scale only under low resource amounts. In addition, among foraging behavior variables, the foraging scale was one of the important foraging behaviors for plant growth. Experimental evidence showed that in comparison to the strategy of allocating more root biomass in nutrient-rich patches, the strategy of growing long roots is more important for nutrient uptake [44]. Our results support this: foraging scale seemed to be more important for plant growth than other foraging behaviors.

Our results might give an insight into how soil spatial configuration affects plant foraging behavior and plant growth and the roles of foraging behavior in whole plant growth under different resource amounts. It should be mentioned that our experiment was relatively short (typically on the order of weeks). Plants with lower resource amounts may especially benefit from a larger foraging scale at the beginning of their life [20]. However, the long-term (across seasons or years) growth of perennial plants involves large amounts of nutrients to compensate for the losses due to root turnover [46]. Thus, further experiments can focus on a longer timeframe. In addition, since plant responses to soil spatial configurations must be considered species-specific, more research is needed to further assess the effects of soil spatial configurations with different resource amounts on other species, population levels and community levels.

## Supporting information

S1 Data Measurements of plant biomass and foraging behavior. (XLSX)
